# What makes domain knowledge difficult? Word usage frequency from SUBTLEX and dlexDB explains knowledge item difficulty

**DOI:** 10.3758/s13428-022-01918-0

**Published:** 2022-08-01

**Authors:** Ulrich Ludewig, Pascal Alscher, Xiaobin Chen, Nele McElvany

**Affiliations:** 1 Center for Research on Education and School Development (IFS), TU Dortmund University, Dortmund, Germany; 2 Hector Research Institute of Education Sciences and Psychology, University of Tübingen, Tübingen, Germany

**Keywords:** Political knowledge, Word frequency, Item difficulty, Statistical suppression effect, Educational assessment

## Abstract

The quality of tests in psychological and educational assessment is of great scholarly and public interest. Item difficulty models are vital to generating test result interpretations based on evidence. A major determining factor of item difficulty in knowledge tests is the opportunity to learn about the facts and concepts in question. Knowledge is mainly conveyed through language. Exposure to language associated with facts and concepts might be an indicator of the opportunity to learn. Thus, we hypothesize that item difficulty in knowledge tests should be related to the probability of exposure to the item content in everyday life and/or academic settings and therefore also to word frequency. Results from a study with 99 political knowledge test items administered to *N* = 250 German seventh (age: 11–14 years) and tenth (age: 15–18 years) graders showed that word frequencies in everyday settings (SUBTLEX-DE) explain variance in item difficulty, while word frequencies in academic settings (dlexDB) alone do not. However, both types of word frequency combined explain a considerable amount of the variance in item difficulty. Items with words that are more frequent in both settings and, in particular, relatively frequent in everyday settings are easier. High word frequencies and relatively higher word frequency in everyday settings could be associated with higher probability of exposure, conceptual complexity, and better readability of item content. Examining word frequency from different language settings can help researchers investigate test score interpretations and is a useful tool for predicting item difficulty and refining knowledge test items.

## Introduction

The design of high-quality assessments for knowledge, abilities, and competencies is a major research topic in educational assessment and psychometrics (American Psychological Association, APA Task Force on Psychological Assessment & Evaluation Guidelines, 2020; Care et al., 2018). A high-quality assessment should be based on a solid theory about the domain, and this theory should be able to explain why items are difficult or easy (Mislevy et al., 2003). Essentially, difficulty is a property of an item that describes how much skill, ability, or knowledge is required to solve the item (Embretson & Reise, 2013). Domain-related and theory-based features of test items should explain item difficulty to allow valid interpretations of test results. Different approaches to identifying domain-related item features have proven successful in various fields. One way to organize these approaches is to divide them into structure-driven, complexity-driven, and exposure-driven approaches.

*Structure-driven* approaches assume that a domain consists of distinct elements (i.e., concepts or skills) that have a defined relationship with one another, and solving an item requires some subset of these elements. This approach has been formally defined in (probabilistic) knowledge space theory (e.g., Stefanutti et al., 2012). For example, Tatsuoka (1990) described the domain of solving fraction problems on the basis of seven elements, termed skills (e.g., distinguishing whole numbers from fractions or converting whole numbers to fractions). The domain structure of fractions is hierarchical, because some skills are prerequisites for other skills (e.g., performing the basic fraction subtraction operation and distinguishing whole numbers from fractions are prerequisites for borrowing one from the whole number to the fraction). Other examples of concise knowledge structures can be found in stoichiometry (mathematical chemistry; Segedinac et al., 2018), stochastic problem solving (Stefanutti et al., 2012), or the laws of mechanics (Reif & Heller, 1982). In concise hierarchical domain structures, items are characterized based on the specific subset of skills required, where students fail to answer items if they lack a skill, and items that require more and higher-order skills are more difficult.

*Complexity-driven* approaches assume that proficiency relates to processing capacity and item difficulty to complexity. Broadly defined, complexity refers to a number of variable elements that must be related to each other to answer an item. In spatial (e.g., Embretson & Yang, 2006) and analogical reasoning (Stevenson et al., 2013), the cognitive complexity of items is defined based on the number and type of cognitive operations (e.g., mentally rotating or mirroring shapes) necessary to relate all variable elements and to falsify or verify response options. In passage comprehension, one measure of complexity is propositional idea density, which assesses the number of propositions that need to be related to answer an item (e.g., Ozuru et al., 2008). In complexity-driven approaches, items are characterized via additive features that contribute to complexity, where students fail to answer items because the complexity exceeds their processing capacity, and items that are more complex are more difficult.

*Exposure-driven* approaches characterize items based on the frequency and intensity of associated learning opportunities. For word recognition, items with infrequent words are more difficult to solve than items with frequent words (e.g., Brysbaert et al., 2019). Politicians who are present in the media are more likely to be known than those who are less present (Westle & Tausendpfund, 2019). Overall presence of information has been found to be a main driver for item difficulty in knowledge tests about authors, newspapers, and television (see the environmental opportunity hypothesis; Stanovich & Cunningham, 1993). Thus, items can be characterized according to measures of exposure frequency or presence; students fail to answer items because they were insufficiently exposed to the underlying content or lack the ability to learn from exposure. Items with content for which there are few opportunities to learn should be more difficult.

In educational psychology and assessment, domain knowledge tests are important for theory development (e.g., Kim et al., 2021) and monitoring educational outcomes (National Research Council, 2012). However, relatively few studies have systematically examined item difficulty on domain knowledge tests. We define domain knowledge as factual (e.g., knowledge of terminology) and conceptual knowledge (e.g., knowledge of theories, models, and structures) relevant to a particular domain (e.g., science or politics). In general, it is plausible to assume that structural features (e.g., some concepts might be on a higher order than others), complexity features (e.g., some concepts might be inherently more complex than others), and exposure features (e.g., some concepts might be more present than others) influence item difficulty in domain knowledge tests.

There are models that describe the structure and complexity of domain-specific knowledge (e.g., science: Kim et al., 2021; politics: Weißeno et al., 2010) and more holistic, domain-general taxonomies (e.g., types and qualities of knowledge: De Jong & Ferguson-Hessler, 1996; Bloom’s taxonomies: Krathwohl & Anderson, 2010). However, these models usually do not state what particular factual or conceptual entities are difficult or easy (e.g., Tauber et al., 2013). Thus, it seems worthwhile to investigate objective indicators explaining the difficulty of items in domain knowledge tests.

The current study developed a new approach to explain the difficulty of items in knowledge tests, focusing on factual and conceptual knowledge in the political domain. Knowledge is mainly conveyed through language; therefore, language use could be an indicator of knowledge item difficulty. Using corpus databases from everyday and academic settings, it is possible to measure how frequently words are used in a given setting. Word frequency could be indicative of knowledge item difficulty, because usage frequency could be related to the likelihood of being exposed to the item content, and relative frequency in everyday settings could be associated with real-life experiences. Word frequency has rarely been applied to assess educational achievement, likely due to a lack of understanding of the relationship between domain knowledge and language use. The present study shows that word frequency can be useful for predicting item difficulty in domain knowledge tests.

## Political knowledge and language

Political knowledge involves the ability to recall from memory facts about a political system (i.e., terminology, theories, or structures) that are relevant for interpreting and understanding happenings and developments within that system (Clark, 2017). As such, political knowledge helps people understand political debates and their relevance, sort and categorize political information, and become aware of their own political needs and preferences and what political actions and decisions must be pursued to satisfy these needs and preferences (Cramer & Toff, 2017).

Political knowledge is mainly conveyed and expressed through language. Domain knowledge can be acquired intentionally through academic activities in school or incidentally through exposure to media and real-life experience (Irwing et al., 2001). Students learn factual and conceptual knowledge about political issues through media consumption (i.e., news, television, radio, and social media: Bischof & Senninger, 2018) as well as through oral and written discourse in everyday life or in the school context (e.g., Carpini & Keeter, 1996). There are different ways to learn political facts and concepts, but they are all primarily conveyed through language.

We hypothesize that two major aspects might influence the difficulty of political knowledge items and items in other knowledge domains tightly linked to language use. First, how present is the particular topic in people's lives? Most people know more about present and salient issues (i.e., that people speak, hear, and read about) in their lives (e.g., what democratic institution makes laws that directly affect people’s lives) than those that are rarer (e.g., certain laws that only apply in exceptional situations).

Second, in what settings are these issues present? Some are more present in everyday life (i.e., knowing about one’s country’s current head of government) than in academic settings. Other issues are more present in academic settings (i.e., the structure of the separation of powers) than in everyday life.

In language research, word frequency has been considered an indicator for the probability of exposure and used to explain the difficulty of test material. It is also considered important to distinguish between different language use settings.

## Word frequency and exposure

Word usage frequencies have impressive explanatory power for many language-related tasks. Visual word recognition is slower and more likely to be incorrect when words are used infrequently (Brysbaert et al., 2018). Word frequency is associated with accuracy and latency in semantic classification tasks (Taikh et al., 2015). Infrequent words are more often unknown than frequent words (Brysbaert et al., 2019). Texts are more challenging if they contain rarer words (Berendes et al., 2018; Fitzgerald et al., 2015), and word frequency calculated based on different corpora contributes to explaining text complexity (Chen & Meurers, 2018).

There is a complex debate on the underlying causes of the word frequency effect (see Brysbaert et al., 2018, for a more detailed review). One primary and quite intuitive reason for the word frequency effect is that individuals are frequently exposed to words in language use. With repeated exposure, words become more accessible, and it is more likely that individuals associate a distinct meaning with them (Juhasz et al., 2019). Furthermore, research shows that word frequency based on corpora is most representative of everyday language settings and explains performance in word recognition tasks better than word frequencies gathered from less representative settings (Brysbaert et al., 2011). Individuals are exposed to frequent words more often, and the probability of word exposure is associated with the probability and extent of being familiar with words.

Word exposure and exposure to concepts and facts in the political domain are not inevitably connected, but should be associated with one another in authentic situations. On the one hand, students could theoretically be frequently exposed to the word “parliament” in some decontextualized way (e.g., in spelling excesses). Thus, word exposure need not necessarily be an opportunity to learn about the concept of a parliament. On the other hand, concepts and facts about parliaments could be conveyed using a synonym (e.g., “congress” or “legislature”). Thus, exposure to a specific word is not a necessary prerequisite for learning about the concept of a parliament. However, in authentic language use, exposure to words will be embedded in a related context, and learning about concepts will probably involve exposure to different synonym words. In authentic language use contexts, a test item’s word frequency could therefore be a good indicator of exposure to associated concepts and facts.

## Language setting

Exposure in everyday language settings might not be the only relevant aspect related to word frequency. Knowledge is passed on and expressed through academic language. Academic language is the specialized language in academic settings that facilitates communication and thinking about specific content domains (Nagy & Townsend, 2012). Texts that convey knowledge, such as textbooks, lexicons, or encyclopedias, are a good representation of language in academic settings (Coxhead, 2000).

In contrast to word frequency in everyday language settings, word frequency in academic settings could potentially influence knowledge test item difficulty in two ways. On the one hand, words that are frequent in these academic settings are helpful for communicating knowledge and should be more familiar to students who have been exposed to academic content. Thus, words frequent in academic settings could be an indicator for probability of exposure in academic contexts. However, students are much less exposed to language in academic than in everyday settings. Therefore, word frequency in academic settings might be an inferior indicator for the probability of exposure relative to word frequency in everyday language settings (Brysbaert et al., 2011; Coxhead, 2000).

On the other hand, words that are frequent in academic settings might be associated with more academically cultivated language, used in formal definitions of terminology, theories, models, and structures. Thus, controlling for word frequency in everyday settings (i.e., the best indicator for exposure), word frequency in academic settings could explain additional variance in item difficulty by capturing the degree to which the item content is divorced from everyday language use.

In sum, word frequencies could be beneficial for determining difficulty in knowledge tests, such as those for political knowledge, because they could be an indicator for probability of exposure to the item content and/or the degree of academic cultivation. Presumably, one of the best indicators of exposure is word frequency in everyday life settings. Word frequency in language settings used to convey knowledge (i.e., academic language) could additionally contribute to explaining item difficulty. On the one hand, it could be a *congruent* indicator of exposure in academic settings; on the other hand, it could be a *complementary* indicator that captures the content’s degree of formal and academic sophistication as indicated by its divergence from word frequency in the everyday setting.

## The present study

Understanding what features make domain knowledge tests difficult is vital for supporting an evidence-based test result interpretation. Word frequency can be viewed as a proxy for exposure probability. Word frequency might be an indicator for the frequency of opportunities to learn about political concepts and facts in everyday life and academic settings. The frequency of opportunities to learn should be a main driver of knowledge item difficulty.

Considered separately, does word frequency in everyday and academic settings have a *congruent* effect on explaining the difficulty of items in political knowledge tests?

- H1: Average word frequency in everyday and academic settings individually explains item difficulty
  - H1a: Average word frequency in everyday settings is negatively associated with item difficulty (low word frequency is associated with more difficult items)
  - H1b: Average word frequency in academic settings is negatively associated with item difficulty (low word frequency is associated with more difficult items)

Considered together, does combining word frequencies from everyday and academic settings in one analysis have a *complementary* effect?

- H2: Combining average word frequency from everyday and academic settings leads to a significant increase in the explained variance in item difficulty.

## Methods

### Participants

Seventh- and tenth-grade students from German schools in mid-sized cities participated in a study on the development of political and civic competencies among youth in fall 2019. In total, 152 seventh graders (*M*_*age*_ = 12.54, *SD* = 0.91, range = 11–14 years; 45% female) and 98 tenth graders (*M*_*age*_ = 16.12, *SD* = 0.97, range = 15–18 years; 35% female) participated in the study. The sample included students from four types of German public schools: “Hauptschulen” (lower vocational track), “Realschulen” (vocational track), “Gymnasium” (academic track), and “Gesamtschulen” (comprehensive schools). Among participants, 43.6% had an immigrant background (i.e., either one or both parents were born outside of Germany). This proportion of students from immigrant backgrounds is higher than the national average in Germany but common for urban West Germany. Additionally, the proportion of females in the tenth grade was significantly below 50% because one of the tenth-grade schools had a vocational orientation that primarily attracted male students. Thus, the sample captures the variability in achievement levels among German students but is not representative. However, unbiased estimates of item difficulties can be derived from unrepresentative samples (Embretson & Reise, 2013; implications discussed in the limitations). The responsible ethics committee approved the study, and all participants or their parents (if the students were younger than 16) were asked to give their informed consent. Only participants with valid informed consent forms were allowed to participate in the study.

### Materials

#### Political knowledge test

##### Items

The political knowledge assessment was developed for a national large-scale assessment study and included 99 items covering different aspects and facets of political knowledge (Alscher et al., 2022). The items were constructed in a workshop with five content experts and one test administration expert. The items were initially constructed by a team led by a political scientist; then, the experts reviewed all items independently in terms of factorial correctness, solvability, and grade appropriateness. Additionally, the experts revised the items independently regarding language use. The goal was to create items with suitable and authentic language that was as simple as possible and as complex as necessary.

**Fig. 1:**
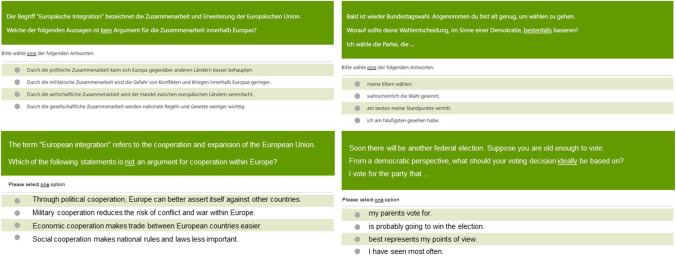
Example items in original German (top) and English (bottom). Items 51 (left) and 29 (right)

The 99 items included 36 grade-specific items and 27 anchor items that students from both grades answered. All items were multiple-choice with four answer options, only one of which was correct (See Fig. 1). The items consisted only of text (i.e., no figures or tables), with between 22 and 154 words (prompt, question, answer options). Overall, the test had high reliability (*REL*_eap_ = .89). All items combined included a total of 4819 words (token) and 1569 types (individual or unique words).

##### Item difficulty

Item difficulty is an item trait given by the proportion of students who are capable of answering the item correctly. We applied an effort-moderated (Wise & DeMars, 2006) unidimensional Rasch multi-group IRT model with the TAM package (Robitzsch et al., 2018) in R (R Core Team, 2014) using marginal maximum likelihood to estimate the item difficulty parameter. Instead of point estimates, we used a plausible value approach with ten drawings to enable a measurement error-adjusted and unbiased estimation of effects in further analysis.

#### Word frequency

Word frequency in everyday settings was measured with SUBTLEX-DE (http://crr.ugent.be/archives/534; Brysbaert et al., 2011). SUBTLEX-DE consists of German-language subtitles from 4610 films and television shows, resulting in a corpus of 25,399,040 words (i.e., tokens) and 319,536 different words (i.e., types). In total, only 8.41% of all types in the items were not found in SUBTLEX-DE.

Word frequency in academic settings was measured via the lexical database dlexDB. DlexDB is based on the core corpus of the Digital Dictionary of the German Language (DWDS). The DWDS core corpus is a reference corpus of the German language in the twentieth century, balanced in terms of time and text types, and has the following composition in its online version (fiction: ca. 28%, newspapers: ca. 27%, academic literature: ca. 23%, practical texts: ca. 21%). The core corpus of the DWDS has a volume of ca. 100 million running text words (tokens). The number of different words (types) is approximately 2.3 million. In total, only 1.91% of all types in the items were not found in dlexDB.

We used multiple imputation to address the missing word frequencies. Multiple imputation has been shown to be the least biased method of dealing with missing data (Sinharay et al., 2001). Multiple imputation is most effective when missing values can be imputed based on non-missing information; therefore, it is best practice to include additional variables that are not part of the intended analysis (also called “auxiliary variables”: Mustillo & Kwon, 2015). For the multiple imputation, (1) we gathered auxiliary variables: word frequencies from web texts (541,453,764 tokens; 6,303,178 types; non-found: 0.32%), German Wikipedia in 2021 (https://wortschatz.uni-leipzig.de/de; 17,765,613 tokens; 983,883 types, non-found: 2.42%), the part of speech, and character length, (2) we used the *mice* package (van Buuren & Groothuis-Oudshoorn, 2011), the *norm* method (i.e., imputation via Bayesian linear regression), 200 restarts, and 20 imputed datasets. Multiple imputation should increase the reproducibility of our results (e.g., in other languages) because the variance in items’ average word frequencies and the covariance between word frequencies from different corpora will be less biased by corpus size than with other methods.

We computed so-called Zipf values based on the raw word frequencies with capitalization normalization (Diependaele et al., 2013; Van Heuven et al., 2014). Zipf values are logarithmically scaled, account for the size of a corpus, and are transformed so that a value of 3 corresponds to the frequency of a word that occurs once in a million words, a value of 4 ten times in a million words, a value of 5 100 times in a million words, etc. Finally, we calculated the arithmetic average of all word (i.e., type) frequencies for the analysis on the item level.

We calculated three different average word frequencies: first, a simple average word frequency including all words in items; second, the mean frequency of all words except stop words, using the *R* package *stopwords* (Benoit et al., 2021), to decrease the effect of very frequent function words; and third, the mean frequency of nouns, verbs, and adjectives, because nouns, verbs, and adjectives should best characterize the actual facts and concepts addressed in an item. For the analysis presented herein, we primarily report the average frequency of nouns, verbs, and adjectives (additional analyses can be found in Appendix 2).

### Procedure

The political knowledge assessment was administered as part of a study on civic literacy on 10.1-inch tablets in class settings. The political knowledge assessment was the first part of the study and took 60 minutes. Each student completed the 27 anchor items and 36 grade-specific items. The items were presented in nine different orders. The item orders were permutated block-wise in a Latin square to counterbalance order effects (Frey et al., 2009). The blocks included equal proportions of items from different content areas and anchor items. The test was administered with a forced-choice answer format. To reduce the influence of rapid-guessing behavior, we visually identified the threshold in the response time distribution (following the recommendations of Wise & DeMars, 2006) at 4.5 seconds, leading to the deletion of 2.48% of responses. In the end, together with non-reached items, a total of 5.71% of all responses were missing. The full study took 3 hours. Besides the knowledge assessment, students were asked to answer demographic and political orientation questions.

### Analysis

#### Presented analysis

For the analysis, first, we used an ordinary least squares (OLS) regression using the *lm* function from the *base* package (R Core Team, 2014) to explain the item difficulty parameters based on the (1) average word frequency in everyday settings alone, (2) average word frequency in academic settings alone, and (3) both word frequencies simultaneously. All results are based on coefficients pooled from 20 consecutive analyses using the 20 imputed datasets with plausible item difficulty values. Second, we calculated the significance of changes in (pooled) coefficient size between 1 and 3 or 2 and 3, which indicate mediation or suppression effects, respectively, according to MacKinnon et al. (2000). Third, we present a regression based on orthogonal principal component analysis using the *prcomp* function from the *stats* package (R Core Team, 2014).

According to G*Power (Faul et al., 2007), given 99 observations (items), *p*-value *p* < .05, two predictors, and 80% test power, the analysis has an ability to detect a medium effect *f*^2^ ≥ .10 (*R*^2^ = .091). For the *R*^2^ increase (*p* < .05, power = 80%) from a one-predictor model to a two-predictor model, the detectable effect is *f*^2^ ≥ 0.081 (*partialR*^*2*^ = .074). The analysis was not preregistered. The data and scripts are available at https://osf.io/bsn9m/.

#### Robustness analysis

Word frequencies in different settings are highly correlated, and under some conditions, multicollinearity can cause computational problems (Cohen et al., 2003). First, we calculated the variance inflation factor (VIF) for the combined model. The VIF ranged between VIF = 2.91 and 3.10 and thus did not indicate problematic multicollinearity (critical VIF > 5; Akinwande et al., 2015). Second, we validated that the correlation matrix was non-negatively defined (Friedman & Wall, 2005). Third, we replicated the results using resampling methods with the *train* function from the *caret* package (Kuhn, 2021), applying the method “repeatedcv” (with ten repetitions of fivefold cross-validation) and “boot632” (with 1000 bootstraps). The regression weights and *R*^2^ estimates from the resampling methods did not deviate from the original OLS regression. Fourth, we applied a ridge regression using the *glmnet* function from the package with the same name (Friedman et al., 2010) to validate the *R*^2^ of the combined model. The ridge regression did not yield a different *R*^2^. Fifth, we replicated the estimates with the formula presented by Friedman and Wall (2005; formula and results can be found in Appendix 3). The results did not deviate from the OLS regression. Sixth, we calculated the results using residualized variables for academic and everyday word frequency (Wurm & Fisicaro, 2014; formula and results can be found in Appendix 3). The robustness analyses did not yield different estimates or different interpretations from the presented analysis.

## Results

### Preliminary results

**Fig. 2:**
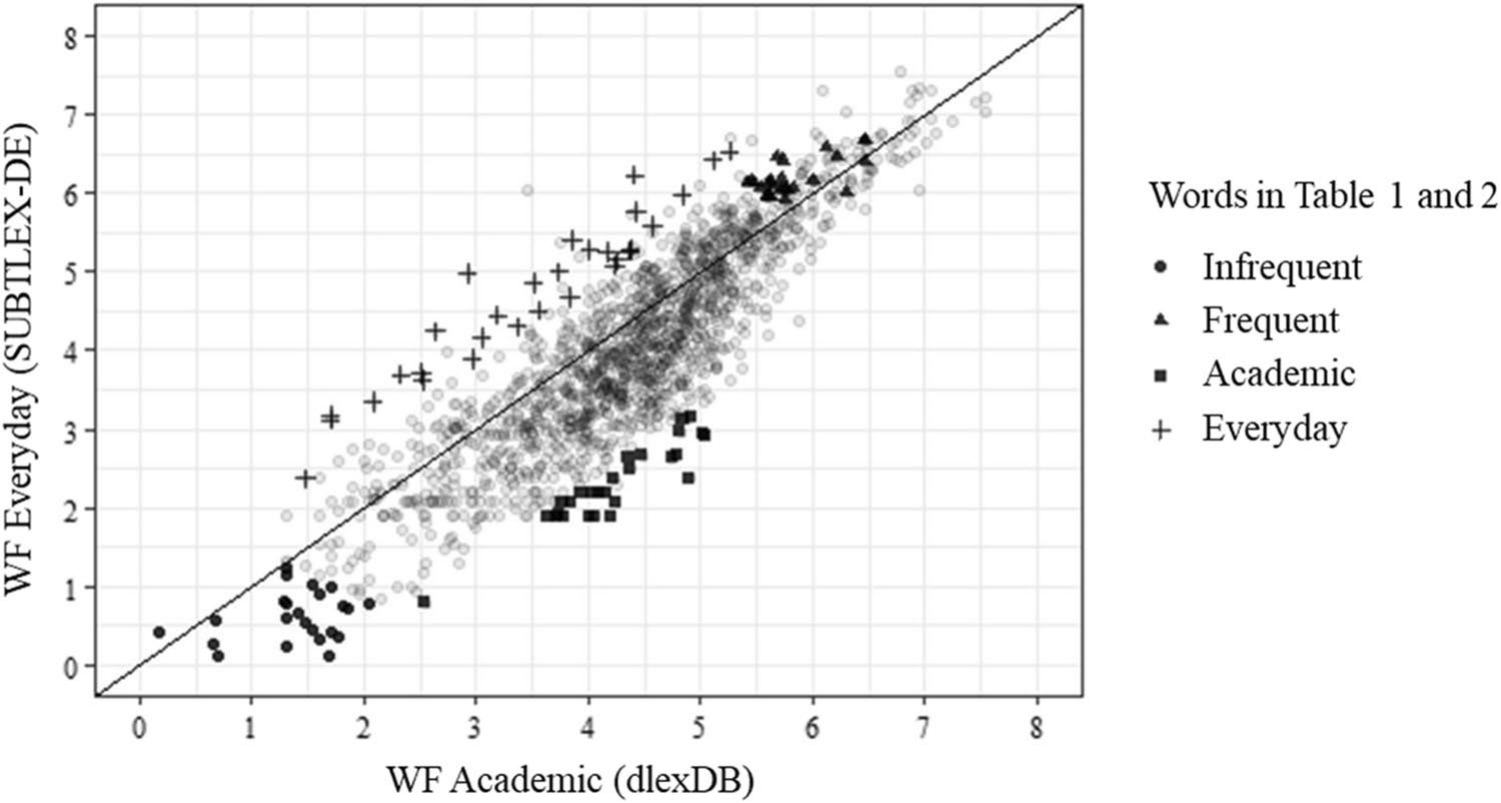
Bivariate distribution of word frequency in everyday and academic settings. *Note.* Words above the diagonal are relatively more frequent in academic settings and words below the diagonal are relatively more frequent in everyday settings

Figure 2 shows the bivariate distribution of word frequency in academic and everyday settings. The two word frequencies are highly correlated, *r*(1, 567) = .82 (without stop words: *r* = .76; only nouns, verbs, and adjectives: *r* = .77). To illustrate which words are frequent, infrequent, relatively more frequent in everyday settings, and relatively more frequent in academic settings, we display the top 30 words for each setting in Tables 1 and 2. The most frequent words are mostly auxiliary verbs. Words that are relatively more frequent in everyday settings include colloquial terms (e.g., bescheuert [stupid] or entschuldigen [saying sorry]) and terms that are related to students’ life situation (e.g., Schulabschluss [graduation], Klassenstufen [grade], or Vorstellungsgespräch [job interview]). The infrequent words are mostly nouns specific to political topics. Many of them are composite words. Words that are relatively more frequent in academic settings encompass fewer composite words, and more key terms related to the topic of politics (e.g., Bundesrepublik [Federal Republic]).

**Table 1:** Top 30 most frequent (see Fig. 2; triangles) words (nouns, verbs, and adjectives) and most infrequent words (circles)

	Frequent		Infrequent	
	German	English	German	English
1	hat	has	Regierungsvorsitzenden	government chair
2	sind	are	Ausgangsbeschränkungen	output restrictions
3	wird	will	Umweltvereins	environmental association
4	habe	have	Arbeitnehmerschutzes	worker protection
5	kann	can	Spendenquittungen	donation receipts
6	hatte	had	Supranationalitätsprinzip	supranationality principle
7	können	can	Pflichtversicherungen	Compulsory insurance
8	meine	my	Nationenprinzip	principle of nations
9	gut	well	Hoheitsprinzip	sovereignty principle
10	machen	make	Konfliktprinzip	conflict principle
11	soll	should	Gleichbehandlungsgesetz	equal treatment law
12	gibt	gives	Asylberechtigung	right of asylum
13	leben	live	Fachministerin	specialized minister
14	müssen	must	Kollegialitätsprinzip	principle of collegiality
15	sagen	say	Gesetzesvorschlägen	legislative proposals
16	viel	much	Kanzlerprinzip	hancellor principle
17	geht	goes	Ressortprinzip	departmental principle
18	hast	have	Menschenrechtsverstöße	human rights violations
19	vielleicht	maybe	Klassensprechern	class representatives
20	lassen	let	EU-Ebene	EU-level
21	kommt	comes	Anführerinnen	leaders
22	tun	do	Bundeländern	federal states
23	gehen	go	Diktatorin	dictator
24	wissen	know	Verursacherinnen	causers
25	macht	power	Verbrecherinnen	criminals
26	kommen	come	Luxusreisen	luxury travel
27	wollte	wanted	Politikunterricht	politics lessons
28	wollen	want	Strafverfolgungen	prosecutions
29	wirklich	really	Anwohnerinnen	residents
30	bist	are	Schulsprecher	head boy

*Note.* We classify the words into most frequent, most infrequent, academic, and everyday lists to provide illustrative examples. The classification had no influence on further analysis and is not mutually exclusive (e.g., words in the everyday list can be in the most frequent list)

**Table 2:** Top 30 words (nouns, verbs, and adjectives) more frequent in everyday settings (see Fig. 2; crosses) and more frequent in academic settings (squares)

	Everyday		Academic	
	German	English	German	English
1	passt	fits (slang)	Bundesrepublik	federal republic
2	muss	must	Parlamentarischen	parliamentary
3	bescheuert	stupid	Regierungsvorsitzenden	president of the government
4	solltest	should	Staatspräsident	president of the Republic
5	Schulabschluss	school graduation	Bundeskanzlers	chancellor (possessive)
6	Klassensprecher	class president	Deutschlands	germany’s
7	Reporterin	reporter	Grundgesetzes	constitution (possessive)
8	müsste	should	Regelung	regulation
9	passiert	happens	Bundeskanzler	federal chancellor
10	bist	are	wirtschaftlichen	economic
11	lässt	lets	Wirtschaftspolitik	economic policy
12	hast	have	bayerische	bavarian
13	Klassenstufen	grades	Prinzipielle	principle
14	möchtest	would like	bestehende	existing
15	müssten	would have to	Entfaltung	development
16	bittest	ask	Gesellschaftlichen	social
17	kannst	can	Herrschende	ruling
18	abschalten	switch off	Zielsetzungen	objectives
19	Vorstellungsgespräch	job interview	Spendenquittungen	donation receipts
20	entschuldigen	sorry	Bundesverfassungsgericht	federal constitutional court
21	stimmt	true	Institutionen	institutions
22	Anführer	leader	Pflichtversicherungen	compulsory insurance
23	verschwenden	waste	Neuwahlen	new elections
24	Chefin	boss	Bundesregierung	federal government
25	gefällt	like	hochgebildeter	highly educated
26	kriegen	get	Verflechtung	interconnectedness
27	Telefon	phone	Sozialpolitik	social policy
28	Polizistinnen	policewomen	Solidarität	solidarity
29	passieren	pass	wirtschaftliche	economic
30	aufpassen	watch	Völkerrecht	international law

*Note.* We classify the words into most frequent, most infrequent, academic, and everyday lists to provide illustrative examples. The classification had no influence on further analysis and is not mutually exclusive (e.g., words in the everyday list can be in the most frequent list)

### Descriptive results

Average word frequency was *M* = 4.15, *SD* = 0.35 in everyday settings and *M* = 4.49, *SD* = 0.26 in academic settings. Average word frequency in the two settings was highly correlated, *r*(97) = .77, *p* < .001. Across different measures, the average word frequency in everyday settings was significantly correlated with item difficulty, *r*(97) = −.36, *p* < .001, whereas word frequency in academic settings was not, *r*(97) = −.09, *p* = .352 (see Table 3).

**Table 3:**
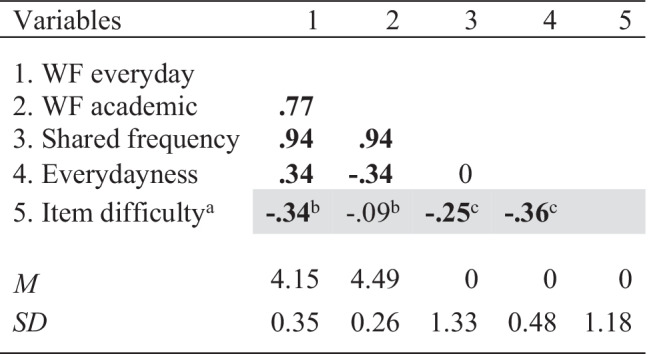
Correlations and descriptive statistics

*Note. N* = 99 political knowledge items. Bold correlations are significant (*p* < .05). WF: Average word frequency of nouns, verbs and adjectives. Light grey fields: Correlations with item difficulty. ^a^Please find histogram displaying the distribution of item difficulty in Appendix 1. Comparison of correlations from dependent samples: ^b^Correlations significantly different, *z* = 9.19, *p* < .001, ^c^Correlations not significantly different, *z* = 1.51, *p* = .065

In addition, we performed a principal component analysis to obtain two orthogonal components. The first principal component was positively correlated with everyday and academic word frequency, *r*(97) = .94, *p* < .001, so we called it *shared frequency*. The second principal component was positively correlated with everyday word frequency, *r*(97) = .34, *p* = .001, and negatively correlated with academic word frequency, *r*(97) = −.34, *p* = .001, so we termed it *everydayness*. Shared frequency, *r*(97) = −.23, *p* = .023, and everydayness, *r*(97) = −.36, *p* < .001, were negatively correlated with item difficulty.

There were slight differences in average word frequency including all words, without stop words, and only considering nouns, verbs, and adjectives with regard to the mean, standard deviation, and correlations that were not statistically significant. The overall average word frequency was lowest when stop words were excluded. The standard deviation was highest when we only considered nouns, verbs, and adjectives (results can be found in Appendix 2).

### Does word frequency in academic and everyday settings explain item difficulty in political knowledge assessment?

We present the regression results for average word frequency using only nouns, verbs, and adjectives in the results section. Please find the equivalent analyses for average word frequency with all words and without stop words in Appendix 2 Table 6. The core findings do not deviate between different average word frequencies.

#### Considered separately, is there a congruent effect?

Word frequency in everyday settings had a statistically significant effect on item difficulty, β_1_ = −0.34, *t* = −3.51, *p* <.001. Items become more difficult as word frequency in everyday settings drops, supporting H1a. Word frequency in everyday settings explains 13% of the variance in item difficulty. In contrast, word frequency in academic settings has no significant effect, β_2_ *= −*0.09, *t* = −0.91, *p* = .364. Thus, Hypothesis H1b is not supported by the analysis.

#### Considered together, is there a complementary effect?

We evaluate the relative contribution of average word frequency from everyday and academic settings to explaining difficulty by entering them both in the regression analysis. In the combined model, both word frequencies have an effect on item difficulty (see Table 4). The regression coefficient for academic word frequency increases when everyday word frequency is entered into the model. Thus, in the combined model, academic word frequency has a statistically significant effect, β_2_ *=* 0.41, *t* = 2.81, *p* = .007. This effect is significantly larger than the effect in the model including only academic word frequency, *SE*_ab_ = 0.12, *t* = −4.26, *p* < .001, and a * b * c’ = −0.22. Additionally, the regression coefficient for everyday word frequency increases when academic word frequency is entered into the model, *SE*_ab_ = 0.12, *t* = −2.68, *p* = .012, a * b * c’ = −0.22. This statistical phenomenon is known as a mutual suppression effect (MacKinnon et al., 2000) or enhancement effect (Friedman & Wall, 2005).

**Table 4:** Regression analysis explaining item difficulty in the political knowledge test

	Only everyday			Only academic			Both together		
	β	*t*	*p*	β	*t*	*p*	β	*t*	*p*
Dependent variable: Item difficulty									
β_1_ WF Eday	**−0.34**	−3.51	<.001				**−0.65**^a^	−4.50	<.001
β_2_ WF Acad				−0.09	−0.01	.364	**0.41**^a^	2.80	.006
*R*^2^	**.12**			.01			**.19**		

*Note.* *N* = 99 political knowledge items, pooled coefficient from 20 imputed datasets, β = standardized regression coefficient. WF: average word frequency of nouns, verbs, and adjectives; bold coefficients are significant (*p* < .05). Appendix 2 includes the analysis with different word exclusion criteria. ^a^βs significantly different in value, *F*(1, 96) = 6.59, *p* = .012

**Fig. 3:**
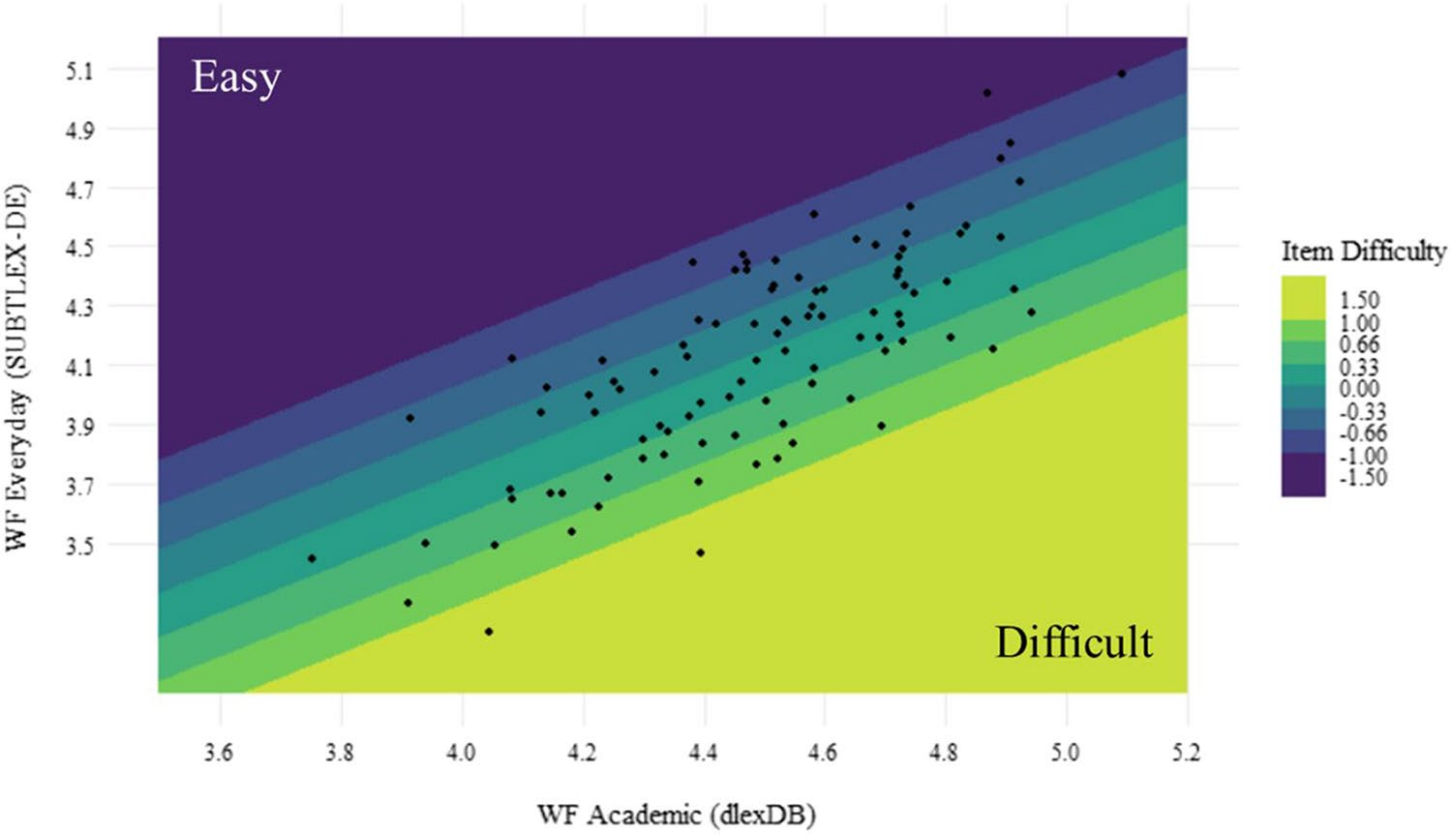
Predicted item difficulty relative to average word frequency in everyday and academic settings. *Note. x*-axis: average word frequency of nouns, verbs, and adjectives from dlexDB, *y*-axis: average word frequency of nouns, verbs, and adjectives in SUBTLEX-DE, points represent the *actual* bivariate distribution of items’ average word frequency. The color represents the *predicted* item difficulty

The model revealed a complimentary effect of combining word frequency in everyday and academic settings. The results suggested that the unshared variance of academic and everyday word frequency explains a substantive additional amount of variance in item difficulty. In other words, item difficulty is explained by relative frequency in each setting respectively. Items with words that are particularly frequent in everyday relative to academic settings are easier. Conversely, items with words that are relatively more frequent in academic settings than in everyday settings are more difficult (see Fig. 3).

**Fig. 5:**
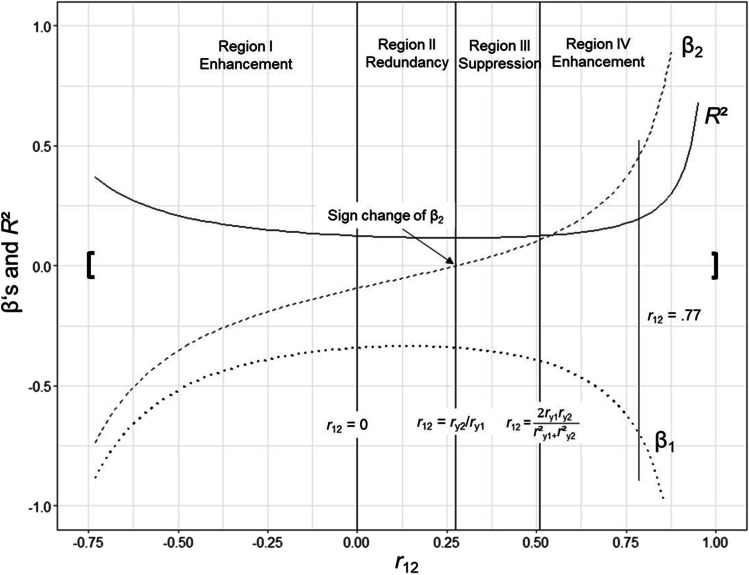
Graphical display of βs and *R*^2^ given the correlation between everyday word frequency and item difficulty (*r*_y1_ = −.34), academic word frequency and item difficulty (*r*_y2_ = −.09), and all possible values for the correlation between everyday and academic word frequency (*r*_12_). *Note*. βs and *R*^2^ for *r*_y1_ = −.34, *r*_y2_ = −.09, and all possible values of *r*_12_. The sign change (i.e., negative correlation *r*_y2_ results in positive β_2_) occurs under the conditions *r*_12_ > *r*_y2_/*r*_y1_ (*r*_y2_/*r*_y1_ = .33). Given the correlations *r*_y1_ = −.34 and *r*_y2_ = −.09, a sign change will occur if the word frequencies are correlated *r*_12_ > .26. Exact estimates for correlations with nouns, verbs, and adjectives: *r*_y1_ = −.3415696, *r*_y2_ = −.09357449, *r*_12_ = .7666544

These results were replicated using regression with residualized variables and the formulas suggested by Friedman and Wall (2005). These results can be found in Appendix 2 Tables 6 and 7, and conditions under which sign changes occur in suppressions in Appendix 3 Fig. 5.

Additionally, we can illustrate these results with the principal components, shared frequency, and everydayness (see Table 5). Item difficulty was explained by the shared frequency component, β_1_ = −0.24, *t* = −2.30, *p* = .024, and the everydayness component, β_2_ = −0.36, *t* = −3.78, *p* < .001. Items were easier when words were frequent in both settings, while the relative frequency in everyday settings (i.e., everydayness) explains additional variance.

**Table 5:** Regression analysis explaining item difficulty in the political knowledge test with principal components of everyday and academic word frequency

	Shared frequency			Everydayness			Both together		
	β	*t*	*p*	β	*t*	*p*	β	*t*	*p*
Dependent variable: Item difficulty									
β_1_ Shared frequency	**−0.24**	−2.30	.024				**−0.24**^a^	−2.46	.016
β_2_ Everydayness				**−0.36**	−3.78	<.001	**−0.36**^a^	−3.88	<.001
*R*^2^	**.05**			**.13**			**.19**		

*Note.* *N* = 99 political knowledge items, pooled coefficient from 20 imputed datasets, β = standardized regression coefficient. Bold coefficients are significant (*p* < .05). ^a^βs not significantly different, *F*(1, 96) = 1.11, *p* =.295

## Discussion

The present paper investigates whether word frequency could be a potential point of reference for item difficulty in political knowledge assessment. Thus, we analyzed how word frequency in everyday and academic settings explains the difficulty of 99 items in a political knowledge assessment administered to 250 German secondary school students in seventh and tenth grade. The results showed that word frequencies in everyday and academic settings significantly explain item difficulty in political knowledge assessment.

Items with words that are more frequent in academic and everyday settings are easier. Thus, we found an effect of word frequency in knowledge test items. The environmental opportunity hypothesis suggests that exposure to content and opportunities to learn plays an important role in knowledge acquisition (Stanovich & Cunningham, 1993). Word frequency could be an indicator of the likelihood of exposure to the facts and concepts addressed in the item and the language used to express them. However, based on this study, we cannot clearly attribute the effect of word frequency to the likelihood of exposure or learning opportunities. First, frequency could also be related to complexity. Inherently complex topics (e.g., European integration) are less likely to be part of informal everyday language use. Therefore, frequently occurring facts and concepts might be intrinsically easier to understand, learn, and express. Second, words with low frequency tend to be longer (Table 1: e.g., Bundesverfassungsgericht “[Federal Constitutional Court]”) and more similar to each other (Table 1: e.g., “Bundestag,” “Bundesrat,” “Bundesamt”). Both word length (i.e., syllable length) and similarity (i.e., OLD20) are factors that reduce readability (e.g., Fitzgerald et al., 2015). Readability is a validity issue if we assume that an item’s decoding demands are higher than its knowledge demands. For instance, there could be students who know something about the Bundesverfassungsgericht [Federal Constitutional Court] but cannot decode the word “Bundesverfassungsgericht”. In sum, the environmental opportunity hypothesis provides an interpretation of the word frequency effect in knowledge tests; however, further research needs to investigate the extent to which complexity, readability, and other factors influence the word frequency effect in knowledge test items.

A novel finding is that combined word frequency in everyday and academic settings has a complementary effect. The relative frequency in everyday settings appears to be very important for explaining item difficulty. Items with words that are relatively frequent in everyday compared with academic settings are particularly easy. It has been argued that word frequency from corpora that best represent the language use to which individuals are actually exposed are the best predictors of difficulty in different tasks (e.g., lexical decision task; Brysbaert et al., 2019). When controlling for word frequency in everyday settings, word frequency in academic settings could become an indicator of the extent to which the item’s content is disconnected from real-life experiences and everyday language use. Item contents that are closer to everyday experiences and everyday language use are easier. However, relative frequency in everyday settings could also be a better indicator for complexity and readability than academic word frequency. These interpretations need to be validated in further systematic research; nonetheless, it seems to be worthwhile to combine word frequency in different language settings when investigating item difficulty.

Overall, our results suggest that word frequency from different language settings can explain item difficulty in educational assessments. An exposure-driven approach led us to assume that word frequency would be an indicator for item difficulty; however, the mechanisms underlying the word frequency effect are probably more complex and multifaceted than merely an effect of exposure and learning opportunities. More research is needed to investigate to what extent word frequency reflects exposure probability, complexity, and readability. Explaining differences in achievement between students and groups of students via differences in opportunities for learning in everyday or academic settings is an area of focus within educational psychology (e.g., Schuth et al., 2017). So there may be several applications where the frequency of words in different contexts could help us better understand why students and groups of students perform differently.

We found that combining highly correlated variables can have a relatively large explanatory value. High correlation does not always mean redundancy (Friedman & Wall, 2005). In this case, we found a suppression effect that we consider theoretically and practically relevant. It should be noted that the results of analyses with highly correlated predictors should be interpreted with caution. Cohen et al. (2003) suggested that suppression effects could be a statistical artifact due to model instability. Contradictory to this, Friedman and Wall (2005) concluded in a study on suppression effects that “our findings indicate that multicollinearity may produce very desirable results” (p. 135). Wurm and Fisicaro (2014) concluded from a study on multicollinearity in psycholinguistic research, “[…] suppression does not indicate computational problems or model instability” (p. 47). The debate on suppression effects could be important because a recent literature review showed that one third of the publications in psychology journals contain evidence of statistical suppression effects (Martinez Gutierrez & Cribbie, 2021). We chose to interpret our results in this manner because the OLS regression with a suppression effect, a regression with orthogonal principal components and several other methods (i.e., cross-validated regression, regression with residualized variables, ridge regression, and Friedman & Wall’s, 2005 formulas) yielded consistent results.

### Strength and limitations

In this paper, we did not build a comprehensive model of item difficulty in political knowledge tests, nor did we examine an exhaustive set of item features. However, word frequency explained 19% of the variance in item difficulty. Word frequencies are very objective, reproducible, and labor-efficient variables and could help to improve item construction. Nonetheless, there is no doubt that other features (e.g., distractor plausibility, structural characteristics of political knowledge) can potentially explain additional variance in item difficulty.

Word frequency is an indicator for exposure *and* often used as a measure of linguistic complexity. On the one hand, conveying niche political facts and concepts will naturally require the use of words with rare frequencies. On the other hand, item difficulty could be influenced by inauthentic and inappropriate use of rare words, making the items overly complex. Our interpretation rests on the assumption that the language in knowledge test items is appropriate. Unfortunately, word frequency does not separate appropriate from inappropriate language use. Appropriateness and authenticity are matters for human judgment. The items were constructed based on international item construction guidelines (Gierl et al., 2017) and reviewed multiple times by experts independently with the objective of ensuring an appropriate level of language complexity. Thus, we have reason to believe that the items use appropriate and authentic language.

The corpora used to determine word frequency in academic and in everyday settings stem from different modalities and are relatively old. The word frequency in everyday settings better captures language exposure via listening, while the word frequency in academic settings better captures language exposure via reading. This modality shift is not generally in conflict with the language setting because everyday language is mostly cultivated through oral communication and academic language is more frequently cultivated through written language. However, a corpus of academic language in oral settings would allow for the more straightforward interpretation of our results. In addition, most of the language sources in both corpora are older than 10 years, and students between the ages of 11 and 18 are unlikely to have consumed them when they were published. Unfortunately, we do not know of a corpus in German that would be more suitable for this analysis.

The number of participants was relatively small compared with other studies in educational assessment. However, Rasch models are usually applicable in studies with smaller sample sizes (e.g., Stone & Yumoto, 2004). Additionally, we used a plausible value procedure to account for methodological issues caused by uncertainty in point estimates (e.g., Marsman et al., 2016). Nonetheless, the results should be replicated in future studies.

The study is based on a non-representative sample of students. However, a core assumption in item response theory is that unbiased estimates of item properties can be obtained from unrepresentative samples (Embretson & Reise, 2013). Therefore, the results concerning item difficulty should be largely reproducible in representative samples. Nonetheless, possible differential item functioning (e.g., Holland & Wainer, 2012) between students from different family backgrounds, for instance, in items with more everyday and academic content, would undoubtedly be a very interesting research topic. However, this study’s statistical power is too limited to detect such effects. Therefore, this is another aspect that should be investigated in future research.

### Conclusion

People know little about the things they have little to do with. What is striking, however, is the fact that word frequencies seem to provide a simple way to describe exposure to and academic orientation of an item. Thus, word frequencies may be a fruitful indicator to improve our understanding of educational assessment in different language-related domains, not just language testing. We publish our analysis scripts, including item parsing, multiple imputation of non-found types, and plausible value drawing. SUBTLEX and corpora similar to dlexDB exist for many different languages. We encourage other researchers to replicate the analysis for different languages and knowledge domains.

## Figures and Tables

**Fig. 4:**
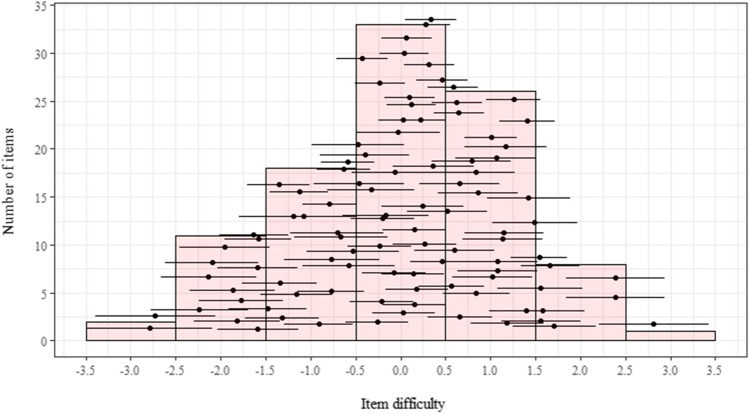
Distribution of item difficulty. *Note. X*-axis scale of item difficulty ranging from −3.5 to 3.5 on a logit scale. Points represent mean difficulty estimate for each item. The range around the points represents the 95% confidence interval of the item difficulty estimate. The *y*-axis represents the number of items within a binwidth of 1. Point position is based on a frequency count within the binwidth and random variation (jitter) to avoid overlapping confidence intervals

**Table 6:**
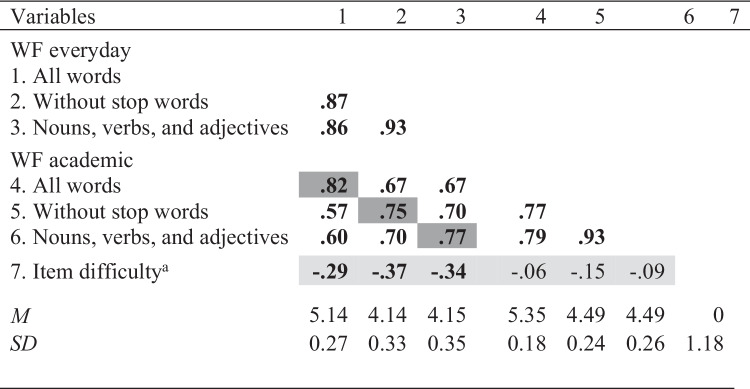
Correlations and descriptive statistics

*Note*. *N* = 99 political knowledge items. Bold correlations, *p* < .05. WF: Average word frequency. Dark grey field: Correlations between everyday and academic word frequency with the same criteria. Light grey fields: Correlations with item difficulty. ^a^Please find histogram displaying the distribution of item difficulty in Appendix 1

## Appendix 1

Appendix Fig. 4

## Appendix 2

Appendix Tables 6 and 7.

**Table 7:** Regression analysis explaining item difficulty in the political knowledge test with different word exclusion criteria

	Only everyday			Only academic			Both together		
	β	*t*	*p*	β	*t*	*p*	β	*t*	*p*
All words									
β_1_ WF Eday	**−0.29**	−2.91	.005				**−0.72**	−4.41	<.001
β_2_ WF Acad				−0.06	−0.58	.567	**0.53**	3.23	.002
*R*^2^	**.08**			<.01			**.18**		
Without stopwords									
β_1_ WF Eday	**−0.37**	−3.85	<.001				**−0.59**	−4.17	<.001
β_2_ WF Acad				−0.15	−1.43	.157	**0.29**	2.08	.041
*R*^2^	**.14**			.02			**.18**		
Nouns, verbs and adjectives									
β_1_ WF Eday	**−0.36**	−3.71	<.001				**−0.68**	−4.54	<.001
β_2_ WF Acad				−0.12	−1.13	.263	**0.41**	2.74	.007
*R*^2^	**.12**			.01			**.19**		

*Note.* *N* = 99 political knowledge items, pooled coefficient from ten imputed datasets, β = standardized regression coefficient. WF: Average word frequency. Eday: Everyday, Acad: Academic. Bold coefficients are significant (*p* < .05)

## Appendix 3

Appendix Tables 8, 9, 10 and Fig. 5

**Table 8:** Regression analysis explaining item difficulty in the political knowledge test with *residualized academic* word frequency variables

	Only WF everyday			Only *e*(Acad→ Eday)			Both together		
	β	*t*	*p*	β	*t*	*p*	β	*t*	*p*
Dependent variable: Item difficulty									
β_1_ WF Eday	**−0.34**	−3.51	<.001				**−0.34**^a^	−3.63	<.001
β_2_ *e*(Acad→ Eday)				**0.26**	2.64	.010	**0.26**^a^	2.81	.006
*R*^2^	**.12**			**.07**			**.19**		

*Note.* *N* = 99 political knowledge items, pooled coefficient from 20 imputed datasets, β = standardized regression coefficient. WF: Average word frequency of nouns, verbs, and adjectives. Eday: Everyday, Acad: Academic. Bold coefficients are significant (*p* < .05). ^a^βs significantly different, *F*(1, 96) = 22.68, *p* <.001

**Table 9:** Regression analysis explaining item difficulty in the political knowledge test with *residualized everyday* word frequency variables

	Only WF Academic			Only *e*(Eday → Acad)			Both together		
	β	*t*	*p*	β	*t*	*p*	β	*t*	*p*
Dependent variable: Item difficulty									
β_1_ WF Acad	−0.09	−0.91	.364				−0.09^a^	−1.00	.321
β_2_ *e*(Eday → Acad)				**−0.42**	−4.49	<.001	**−0.42**^a^	**−**4.49	<.001
*R*^2^	.01			**.18**			**.19**		

*Note.* *N* = 99 political knowledge items, pooled coefficient from 20 imputed datasets, β = standardized regression coefficient. WF: Average word frequency of nouns, verbs, and adjectives. Eday: Everyday, Acad: Academic. Bold coefficients are significant (*p* < .05). ^a^βs significantly different, *F*(1, 96) = 5.48, *p* <.011

**Table 10:** Computation of βs and *R*^2^ given the correlation (Friedman & Wall, 2005) between everyday word frequency and item difficulty (*r*_y1_ = −.34), academic word frequency and item difficulty (*r*_y2_ = −.09), and between everyday and academic word frequency (*r*_12_ = .77)

1	F1: ${\upbeta}_1=\frac{r_{y1}-{r}_{y2}\ast {r}_{12}}{1-{r}_{12}^2}=-0.65$
2	F2: ${\upbeta}_2=\frac{r_{y2}-{r}_{y1}\ast {r}_{12}}{1-{r}_{12}^2}=0.41$
3	F3: ${R}^2=\frac{r_{y1}^2+{r}_{y2}^2-2{r}_{y1}{r}_{y2}{r}_{12}}{1-{r}_{12}^2}=.19$
4	“[…] there are no limits on multicollinearity in regression on two predictors other than those given by the necessity to have a nonnegative definite matrix” (p. 135) Matrix is nonnegative definite if: F4: ${r}_{y1}\ast {r}_{y2}-\sqrt{\left(1-{r}_{y1}^2\right)}\left(1-{r}_{y2}^2\right)\le {r}_{12}\le {r}_{y1}\ast {r}_{y2}+\sqrt{\left(1-{r}_{y1}^2\right)}\left(1-{r}_{y2}^2\right)=-.90\le .77\le .97$
5	“We suggest that the Regions I-IV, delineated in this article, provide a clear structure by which the correlations involved in a regression on two predictors can be analyzed.” (See Appendix 3 Fig. 5)

*Note.* Exact estimates for correlations with only nouns, verbs, and adjectives: *r*_y1_ = −.3415696, *r*_y2_ = −.09357449, *r*_12_ = .7666544
